# Supplementary material for: Fabrication of Electrospun PLA-nHAp Nanocomposite for Sustained Drug Release in Dental and Orthopedic Applications
Source: Materials (Basel). 2023 May 12;16(10):3691. doi: 10.3390/ma16103691 (PMC10221964; doi:10.3390/ma16103691)
Supplement: Supplementary file 1 [file materials-16-03691-s001.zip › materials-2246004-supplementary.pdf]

Article

Fabrication of Electrospun PLA-nHAp Nanocomposite for Sustained Drug Release in Dental and Orthopedic Applications

Nishat Anzum Kanak [1,2], Md. Shahruzzaman [1], Md. Sazedul Islam [1,3], Makoto Takafuji [4], Mohammed Mizanur Rahman [1] and Sumaya F. Kabir [1,*]

[1] Department of Applied Chemistry and Chemical Engineering, University of Dhaka, Dhaka 1000, Bangladesh
[2] Department of Chemistry, University of Pittsburgh, Pittsburgh, PA 15260, USA
[3] Department of Chemistry and Biochemistry, Florida State University, Tallahassee, FL 32306, USA
[4] Department of Applied Chemistry and Biochemistry, Kumamoto University, Kumamoto 860-8555, Japan
* Correspondence: sumaya.kabir@du.ac.bd

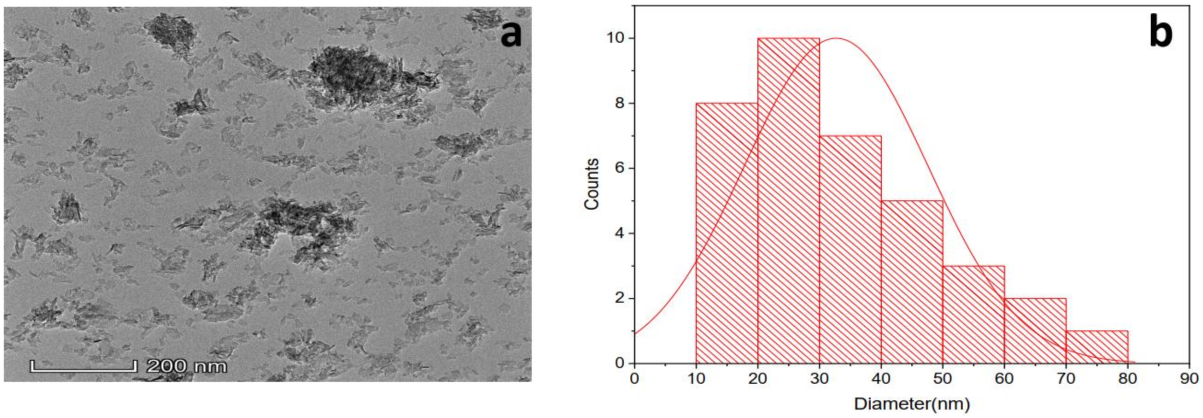

Figure S1. a. TEM image of prepared nHAp; b. Histogram of particle size of nHAp.

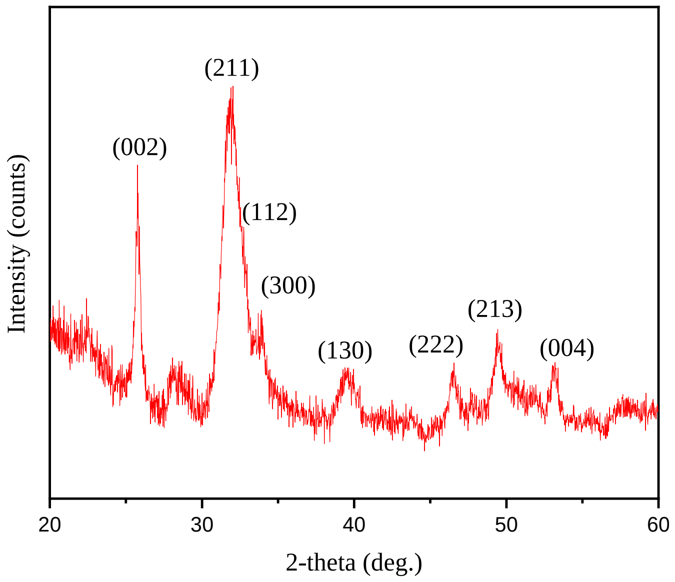

Figure S2. XRD analysis of synthesized nHAp

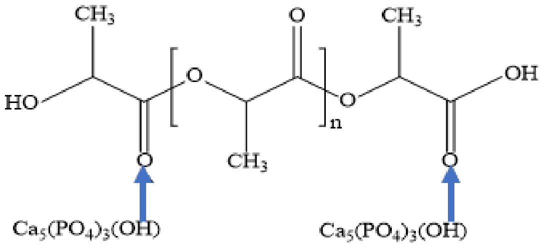

Figure S3 Probable sites for physiochemical bonding between PLA and hydroxyl group of nHAp

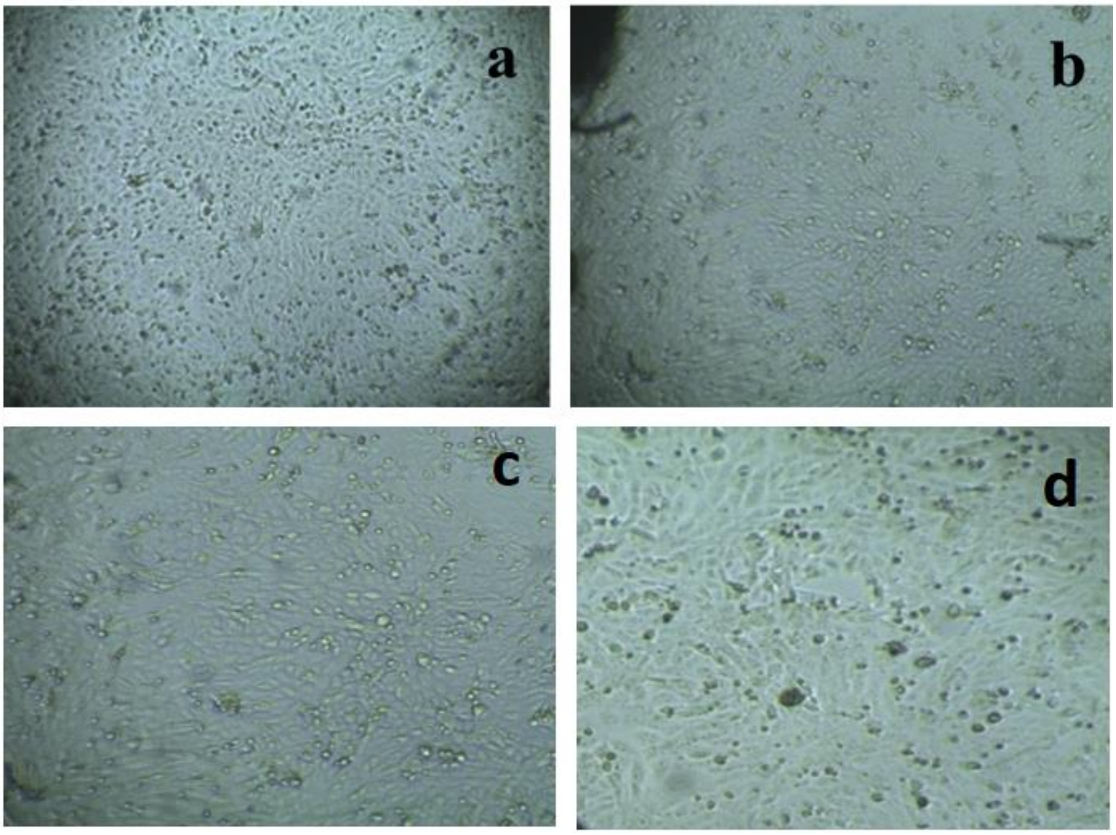

Figure S4. Optical microscopic images of a. Vero cell treated with control; b. Vero cell treated with nanocomposite; c. BHK-21 cell treated with control; and d. BHK-21 cell treated with nanocomposite.

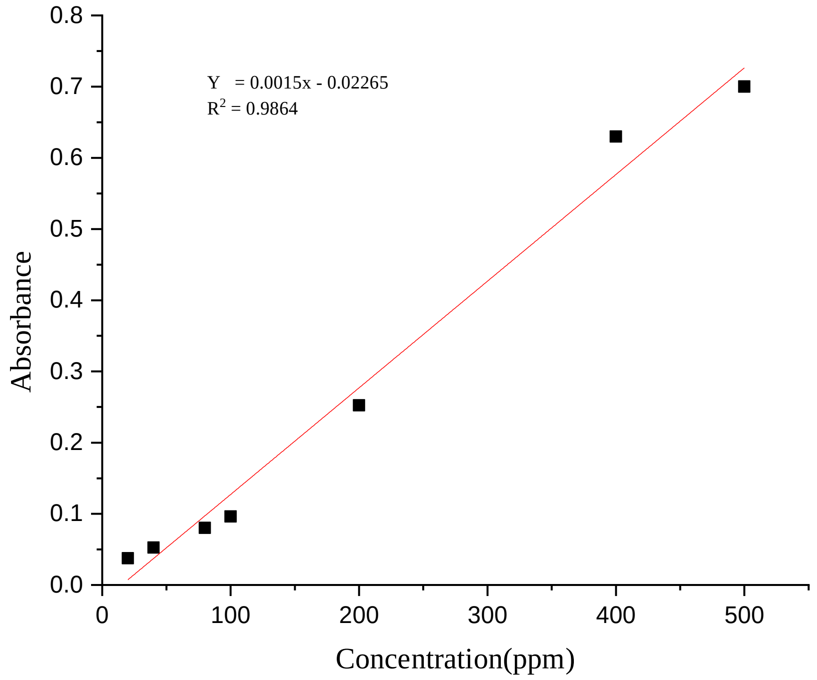

Figure S5. Calibration curve (standard) of Gentamicin sulfate at a wavelength of 400 nm using UV-Visible spectroscopy.
